# Genotypic and Phenotypic Variables Affect Meiotic Cell Cycle Progression, Tumor Ploidy, and Cancer-Associated Mortality in a* brca2*-Mutant Zebrafish Model

**DOI:** 10.1155/2019/9218251

**Published:** 2019-02-26

**Authors:** L. Mensah, J. L. Ferguson, H. R. Shive

**Affiliations:** Department of Population Health and Pathobiology, North Carolina State University, College of Veterinary Medicine, 1060 William Moore Drive, Raleigh, Raleigh, NC, USA

## Abstract

Successful cell replication requires both cell cycle completion and accurate chromosomal segregation. The tumor suppressor BRCA2 is positioned to influence both of these outcomes, and thereby influence genomic integrity, during meiotic and mitotic cell cycles. Accordingly, mutations in* BRCA2* induce chromosomal abnormalities and disrupt cell cycle progression in both germ cells and somatic cells. Despite these findings, aneuploidy is not more prevalent in* BRCA2*-associated versus non-*BRCA2*-associated human cancers. More puzzlingly, diploidy in* BRCA2*-associated cancers is a negative prognostic factor, unlike non-*BRCA2*-associated cancers and many other human cancers. We used a* brca2*-mutant/*tp53*-mutant cancer-prone zebrafish model to explore the impact of* BRCA2* mutation on cell cycle progression, ploidy, and cancer-associated mortality by performing DNA content/cell cycle analysis on zebrafish germ cells, somatic cells, and cancer cells. First, we determined that combined* brca2/tp53* mutations uniquely disrupt meiotic progression. Second, we determined that sex significantly influences ploidy outcome in zebrafish cancers. Third, we determined that* brca2* mutation and female sex each significantly reduce survival time in cancer-bearing zebrafish. Finally, we provide evidence to support a link between* BRCA2* mutation, tumor diploidy, and poor survival outcome. These outcomes underscore the utility of this model for studying* BRCA2*-associated genomic aberrations in normal and cancer cells.

## 1. Introduction

Generation of cell progeny lies at the heart of virtually all biological processes. Successfully performing this fundamental cell behavior requires both completion of the cell cycle and faithful replication and segregation of chromosomal content. Both meiotic and mitotic cell cycles are governed by these principles, although clear mechanistic differences exist (reviewed by Duro E and Marston AL [1]). If cell cycle progression is disturbed during mitotic or meiotic cell cycles, potential adverse outcomes include cell cycle arrest, chromosomal aberrations, and/or missegregation; the latter outcomes may cause chromosomal instability and aneuploidy.

**Figure 1:**
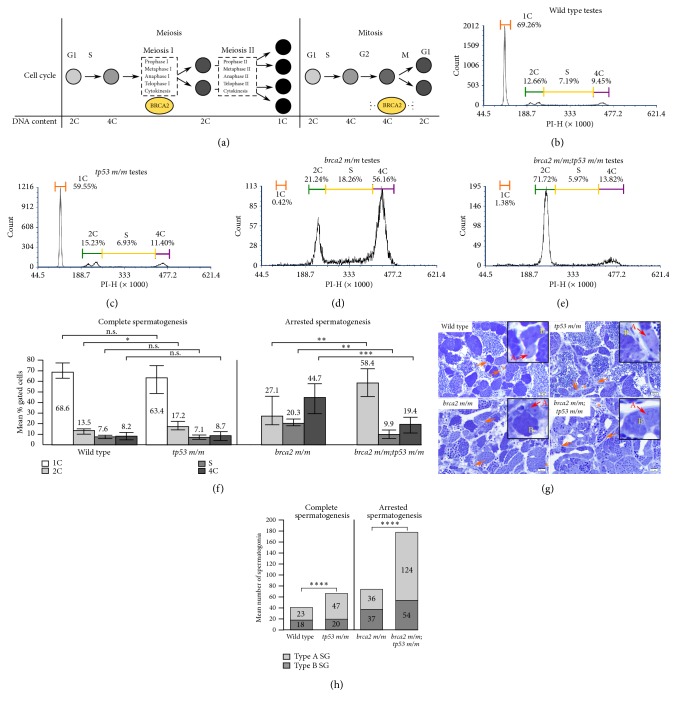
*brca2* and* tp53* mutations alter distribution of cells according to DNA content in adult zebrafish testes. (a) Comparison of cell progression through meiosis and mitosis with corresponding DNA content (designated as 1C, 2C, or 4C). BRCA2 participates in DNA repair during prophase I of meiosis I and performs multiple functions between late G2 and M phases of mitosis, as indicated by the positions of the yellow ovals. ((b)–(e)) Propidium iodide (PI) fluorescence histograms of testes derived from wild type (b),* tp53 m/m* (c),* brca2 m/m* (d), and* brca2 m/m;tp53 m/m* zebrafish testes (e). (f) Mean percent of gated cells clustered by DNA content for wild type,* tp53 m/m*,* brca2 m/m*, and* brca2 m/m;tp53 m/m* zebrafish testes. The mean percent of gated cells for each DNA content category is indicated. (g) Comparison of testicular morphology in wild type,* tp53 m/m*,* brca2 m/m*, and* brca2 m/m;tp53 m/m* zebrafish, Toluidine blue stain. Insets show type A (A, red) and type B (B, yellow) spermatogonia. Orange arrows indicate representative regions of stromal tissue and orange asterisks indicate examples of blood vessels within the stroma. (h) Comparison of the mean number of spermatogonia per 400X field (see Materials and Methods) in wild type,* tp53 m/m*,* brca2 m/m*, and* brca2 m/m;tp53 m/m* zebrafish testes. The mean numbers of type A and type B spermatogonia are shown in the appropriate portion of each column. SG, spermatogonia; *∗*, p = 0.01-0.05; *∗∗*, p = 0.001–0.01; *∗∗∗*, p = 0.0001–0.01; *∗∗∗∗*, p < 0.0001. Error bars represent the range of the data. See Table S1 for specific p-values.

The tumor suppressor gene* BRCA2* functions in multiple pathways that affect both meiotic and mitotic cell cycles, and thereby genomic stability. These include homology-directed repair (HDR), replication fork maintenance, spindle assembly checkpoint (SAC), cytokinesis, and telomere homeostasis (reviewed by Venkitaraman AR [2]).  Figure 1(a) indicates phases of the meiotic and mitotic cell cycles during which BRCA2 is known to function and the corresponding cellular DNA content in each phase. In meiosis, BRCA2 functions in prophase I of meiosis I; cells enter meiosis I with 4C DNA content and exit meiosis I with 2C DNA content following the first meiotic division. In mitosis, BRCA2 participates in multiple processes that span from the G2 checkpoint in late G2 phase to cytokinesis in M phase, as described below. Cells enter G2 phase with 4C DNA content and exit M phase with 2C DNA content.

In mammalian germ cells, loss of functional Brca2 resulted in cell cycle arrest in meiotic prophase I and persistent DNA damage [3, 4]. Additionally, aberrant chromosomal segregation during meiosis was described in* brca2*-mutant* Arabidopsis* gametophytes [5]. In primary somatic cells (mouse embryonic fibroblasts), loss of functional Brca2 caused cell cycle arrest and both structural and numerical chromosomal abnormalities [6, 7]. Additionally, disrupted interaction between Brca2 and the SAC mediator BubR1 resulted in both genomic instability and aneuploidy [8], and BRCA2 deficiency has been linked to defects in cytokineses [9, 10]. BRCA2 may also participate in regulation of entry into mitosis after the G2 checkpoint [11, 12] and was found to be essential for protection of stalled replication forks [13]. These findings indicate that loss of functional BRCA2 severely disrupts both meiotic and mitotic cell cycles and has significant potential to destabilize genomic integrity.

The above studies predict that* BRCA2*-associated human cancers might exhibit a high prevalence of aneuploidy. However, comparison of* BRCA2*-associated and non-*BRCA2*-associated human breast cancers has shown that* BRCA2* mutation does not increase aneuploidy in human cancer [14–16]. Instead, diploid and aneuploid cancers occurred in roughly equal proportions in* BRCA2 *mutation carriers and noncarriers. Moreover, diploidy was identified as an independent negative prognostic indicator for* BRCA2* mutation carriers that was linked to decreased survival time [16]. In contrast, diploidy was a positive prognostic indicator for noncarriers [16]. This observation is at odds with the fact that aneuploidy is generally considered to be a poor prognostic indicator for many human cancers [17–22]. These unexpected findings suggest an unusual and complex relationship between* BRCA2* mutation, ploidy, and survival outcome.

In the current study, we used a zebrafish model to investigate the impact of* BRCA2* mutation on meiotic and mitotic cell cycle outcomes and to assess the relationship between* brca2* mutation, ploidy, and survival in cancer-bearing zebrafish. The zebrafish brca2^Q658X^ mutation is a nonsense mutation that is similar in location and type to pathologic* BRCA2* mutations in humans [23]. The* brca2*-mutant zebrafish line is fully viable [23], unlike most* Brca2*-mutant mouse models (summarized by Evers B and Jonkers J) [24], and thus is useful for* in vivo* studies with adult animals. In human* BRCA2*-associated cancers,* TP53* is frequently mutated, which is thought to be an early and essential step in survival of transformed cells [25–28]. Similarly, we previously showed that the zebrafish tp53^M214K^ mutation [29] exerts a collaborative effect on tumorigenesis in* brca2*-mutant zebrafish [23, 30]. In the current study, we analyzed zebrafish siblings with and without* brca2* mutation on a* tp53*-mutant background, enabling us to assess the specific impact of* brca2* mutation on ploidy outcome.

First, we determined the effect of* brca2* and* tp53* mutations on meiotic cell cycle progression in zebrafish testes by paired flow cytometry and histologic assessment. Second, we determined the influence of* brca2* and* tp53* mutations on ploidy in zebrafish somatic cells and cancer cells and evaluated the contributions of other variables (sex, tumor location) to ploidy outcome. Finally, we identified the individual and combined impacts of* brca2* genotype, sex, and tumor ploidy on survival outcome in cancer-bearing zebrafish.

## 2. Materials and Methods

### 2.1. Zebrafish Study Cohorts

Experiments were performed with adult wild type (AB) zebrafish and adult zebrafish from the brca2^hg5^ and tp53^zdf1^ mutant zebrafish lines, corresponding to the brca2^Q658X^ and tp53^M214K^ mutations, respectively [23, 29]. Mutant alleles for* brca2* and* tp53* are referred to as “m”; individual zebrafish within each genotypic group were siblings. For studies assessing zebrafish with or without brca2^Q658X^ mutation on the tp53^M214K^ background, the compared study populations were composed of siblings. Thus, reference to* tp53 m/m* zebrafish indicates siblings of* brca2 m/m;tp53 m/m* zebrafish that do not carry the brca2^Q658X^ mutation. The study group used for analysis of tumor ploidy was composed of two related cohorts derived from two clutches in order to achieve a target of 50 individuals per genotype. As this target was not achieved with the first cohort, part of a second cohort was included in the study. All animal studies were approved by the Institutional Animal Care and Use Committee, North Carolina State University, Raleigh, NC, and the methods were carried out in accordance with relevant guidelines and regulations.

### 2.2. Zebrafish Husbandry and Genotyping

Zebrafish used in this study were reared in a multirack recirculating containment system. From five to nine days of age, zebrafish larvae received live cultured* Brachionus plicatilis* (L-type rotifers), and, from ten to thirty days of age, zebrafish fry received commercially available, appropriately sized powder diets supplemented with live cultured* Artemia* sp. (brine shrimp). Juvenile and adult zebrafish received commercially available dry zebrafish diets supplemented with live cultured* Artemia* sp. Pathogen testing is performed on a biannual basis with the IDEXX Zebrafish Essential PCR Profile using zebrafish exposed to prefiltration water and swabs of detritus.

Zebrafish were monitored for clinical and gross evidence of tumor development and were collected in chronological order as tumors arose. Zebrafish were humanely euthanized with Tricaine methanesulfonate (300 mg/L) in system water buffered with Sodium Bicarbonate to a pH of ~ 7.0. Live adult zebrafish were genotyped for the brca2^Q658X^ mutation by sequencing over the mutation site as described previously [31]. Zebrafish on the tp53^M214K^ background were maintained as a homozygous mutant line.

### 2.3. Tissue Collection and Histologic Analysis

Normal and tumor tissues were identified and collected by dissection using a stereomicroscope. For DNA content analysis, tissue samples were prepared as described below. For histologic analysis of tumor-bearing zebrafish, a sample of tumor tissue and the coelomic viscera were collected and fixed in 4% Paraformaldehyde. Fixed tissues were routinely processed for decalcification as needed, paraffin embedding, and preparation of hematoxylin- and eosin-stained sections. For histologic analysis of zebrafish testes, fixed tissues were embedded in glycol methacrylate, sectioned at 2.5 *μ*m thickness, and stained with Toluidine blue stain (0.01 g/ml Toluidine blue and 0.01 g/ml sodium tetraborate in distilled water).

Histologic sections were analyzed with an Olympus BX43 microscope and imaged with a DP26 digital camera and cellSens entry microscope imaging software, version 1.5. Histologic images were minimally and globally processed for exposure, contrast, and/or color balance with the GNU Image Manipulation Program, version 2.8.6 (http://www.gimp.org/).

For quantification of spermatogonia in zebrafish testes, three representative images were captured at 400X from the testes of five zebrafish from each genotypic group for a total of 15 histologic sections per genotypic group. For one* brca2 m/m;tp53 m/m* zebrafish, only two representative images were quantified due to insufficient tissue for capturing three high-quality images; thus, a total of 14 histologic sections were evaluated for this genotypic group. Spermatogonia were manually counted in each digital image using the ImageJ Fiji Cell Counter tool [32]. Spermatogonia were identified by histologic characteristics as previously described [33], and type A and type B spermatogonia were counted separately.

### 2.4. DNA Content Analysis

For preparation of dissociated zebrafish testes, both testes from each zebrafish were collected, minced, and incubated in 500 U/ml Collagenase (Collagenase type I in 1X Hank's Balanced Salt Solution in L15 medium) at 28°C for 2 hours with gentle pipetting every 20 minutes. For preparation of dissociated nonneoplastic somatic cells and cancer cells, matched normal and tumor tissues samples from each individual zebrafish were collected and dissociated as described above. Dissociated cells were washed with 1X phosphate-buffered saline (PBS), filtered with a 35 *μ*m filter and fixed with ice-cold 70% ethanol. Cell suspensions were maintained at -20°C for a minimum of 24 hours. After fixation, cell suspensions were washed with 1X PBS and stained with Propidium Iodide staining solution containing RNase (Cellometer PI Cell Cycle Kit, CSK-0112, Nexcelom, Lawrence, MA).

Cell suspensions were analyzed for DNA content with a Beckman Coulter CytoFLEX flow cytometer. The CytoFLEX was maintained and calibrated daily according to the manufacturer's recommendations. Up to 10,000 events were recorded per sample at a flow rate of 10–30 *μ*l/min (up to 300 events/second). Matched normal and cancer specimens from individual zebrafish were analyzed during the same experiment.

Flow cytometry data were analyzed with DeNovo FCS Express 6 Flow Research Edition. For DNA content analysis of testes, the gating strategy was based on the method described by Rotgers et al. [34]. Cell suspensions from zebrafish testes were gated on forward scatter-A (FSC-A) versus FSC-H, followed by gating on Propidium Iodide-A (PI-A) versus FSC-A (Figure S1A). DNA histograms were generated using PI-H, and haploid (1C), diploid (2C), S-phase, and tetraploid (4C) populations were identified as previously described [34]. Percentages of each cell populations identified by DNA content were acquired by defining marker gates for each population (Figure S1A).

For DNA content analysis of cell suspensions from nonneoplastic somatic tissues and cancer tissues, cells were gated on PI-H versus PI-A. DNA histograms were generated using PI-H with the FCS Express 6 Multicycle AV Professional Version (Figure S1B). Cell cycles were modeled with the SL S0 model (sliced nuclei background modeling with zero order S phase). At least 1,000 events were analyzed to generate the cell cycle for all but one nonneoplastic somatic tissue specimen, for which 632 events were analyzed.

### 2.5. Criteria for Exclusion of Samples

DNA content analysis was attempted on testes from age-matched zebrafish in order to meet a target of at least five individuals per genotypic group. Individual results were excluded from the study under the following criteria: insufficient cell number to generate cell cycle profile.

DNA content analysis was attempted on normal and tumor specimens from zebrafish in chronological order, as cancers arose, in order to meet a target of 50 individuals per genotype. Individuals were excluded from the study under the following criteria: (1) found dead; (2) no grossly identifiable tumor tissue; (3) insufficient cell number to generate cell cycle profile; (4) coefficient of variance (CV) of the sample from nonneoplastic somatic tissue > 6.0; (5) inability to define the diploid population in the nonneoplastic somatic tissue specimen.

### 2.6. Calculation of Tumor Ploidy

Tumor ploidy was defined by calculating the DNA index (Table S1) [35]. For tumor samples that contained an internal diploid population with a CV ≤ 6.0, the DNA index was calculated using the G0/G1 peak fluorescence intensity value of the internal diploid population. The internal diploid population was confirmed to be diploid by comparing the G0/G1 peak fluorescence intensity value of this population to the G0/G1 peak fluorescence intensity value of the matched nonneoplastic somatic tissue specimen. For tumor samples that did not contain an internal diploid population or contained an internal diploid population with a CV > 6.0, the DNA index was calculated using the G0/G1 peak fluorescence intensity value of the matched nonneoplastic somatic tissue specimen. Tumors that exhibited multiple peaks were defined as complex aneuploid and a DNA index was not calculated.

For five zebrafish cancers (4* brca2 m/m;tp53 m/m* and 1* tp53 m/m*), an aneuploid population was inconsistently modeled or constituted ≤ 20% of the total population. For eight zebrafish cancers (5* brca2 m/m;tp53 m/m* and 3* tp53 m/m*), a subpopulation of cells was inconsistently modeled as either an aneuploid population or as the G2/M population. For these thirteen cancers, the G0/G1 peak fluorescence intensity value of the predominant diploid population was used to calculate DNA index.

### 2.7. Statistical Analyses

Statistical analyses were performed using JMP Pro 13.2.1 (SAS Institute Inc.). Statistical significance was set at an alpha value of p ≤ 0.05. Comparisons of zebrafish testes were performed between zebrafish exhibiting complete spermatogenesis (wild type and* tp53 m/m*) and incomplete spermatogenesis (*brca2 m/m* and* brca2 m/m;tp53 m/m*). The percentages of cells by DNA content category in zebrafish testes and nonneoplastic somatic tissues were compared by t-test corrected for unequal variances. The four samples from nonneoplastic tissues that exhibited a small aneuploid peak, described above, were excluded from comparison of the percent gated cells in G0/G1, S, and G2/M phases. The numbers of spermatogonia in zebrafish testes were compared by unpaired t-test assuming unequal variances. Comparison of G1 peak PI fluorescence intensity values were tested for normality by fitting a normal distribution and analyzing goodness-of-fit (Shapiro-Wilk W test). Samples from Experiment Seven that exhibited anomalously high G0/G1 peak PI fluorescence intensity values, described below, were excluded from this analysis (5 samples from* brca2 m/m;tp53 m/m* zebrafish, 1 sample from* tp53 m/m* zebrafish). The Chi-square test was used to test for associations in pairwise comparisons of genotype, tumor location, ploidy outcome, and sex. The median survival times were obtained using the Kaplan-Meier test and differences in survival curves were assessed by the log-rank test and Cox's Proportional Hazard Model. Cox's Proportional Hazard Model was used to determine contribution to survival by the purported risk variables (i.e.,* brca2* mutation status, sex, and tumor ploidy).

## 3. Results

### 3.1. Combined* brca2* and* tp53* Mutations Induce Meiotic Arrest and Spermatogonial Expansion in Zebrafish

We previously showed that zebrafish with homozygous brca2^Q658X^ mutation (*brca2 m/m*) develop exclusively as males and exhibit incomplete spermatogenesis with extensive spermatocyte apoptosis, reflecting a conserved role for* BRCA2* in germ cell development [23]. In comparison, homozygous tp53^M214K^ mutation (*tp53 m/m*) was not reported to impact sex ratios or fertility in zebrafish [29]. In the following studies, comparisons were performed between testes with complete spermatogenesis (wild type and* tp53 m/m*) and between testes with incomplete spermatogenesis (*brca2 m/m* and* brca2 m/m;tp53 m/m*).

Similar to* brca2 m/m* zebrafish,* brca2 m/m;tp53 m/m* males are sterile and exhibit incomplete spermatogenesis, with only spermatogonia and primary spermatocytes present in testes (Figure S2A). In comparison,* tp53 m/m* male zebrafish are fertile and exhibit complete spermatogenesis, with no histologic abnormalities observed in testes (Figure S2B). To further investigate this phenotype, we analyzed dissociated testes from age-matched wild type (n = 5),* tp53 m/m *(n = 7),* brca2 m/m* (n = 9), and* brca2 m/m;tp53 m/m *(n = 5) male zebrafish by flow cytometry (Figure 1 and Figure S1A). Testes from wild type and* tp53 m/m* zebrafish had similar cell cycle profiles and exhibited a predominance of cells with 1C DNA content, representing mature spermatozoa (Figures 1(b), 1(c), and 1(f)). The proportions of cells in each DNA content category (1C, 2C, S, and 4C) were not significantly different in testes from wild type and* tp53 m/m* zebrafish, with the exception of the 2C population (p = 0.0405, unpaired t-test; Figure 1(f) and Table S1).

In contrast, testes from* brca2 m/m* and* brca2 m/m;tp53 m/m* zebrafish did not contain an appreciable cell population with 1C DNA content (< 2% of gated cells), indicating arrested spermatogenesis in males from these genotypic groups. Seven of 9 testes from* brca2 m/m* zebrafish exhibited a predominance of cells with 4C DNA content (Figures 1(d) and 1(f)), while all testes from* brca2 m/m;tp53 m/m* zebrafish exhibited a predominance of cells with 2C DNA content (Figures 1(e) and 1(f)). Testes from* brca2 m/m* zebrafish additionally exhibited an increased proportion of cells in S phase. The proportions of cells in each DNA content category (2C, S, and 4C) were significantly different in testes from* brca2 m/m* and* brca2 m/m;tp53 m/m* zebrafish (Figure 1(f) and Table S1).

We next sought to determine a cause for the difference in the proportions of cells with 2C versus 4C DNA content in testes from* brca2 m/m* versus* brca2 m/m;tp53 m/m* zebrafish. The 2C population identified by flow cytometry in dissociated testes includes spermatogonia, secondary spermatocytes, and somatic cells (stromal component). However, secondary spermatocytes are rarely observed in zebrafish testes due to rapid entry into meiosis II [33]. We performed quantitative histologic analysis on thin sections of testes to determine the prevalence of spermatogonia. We analyzed testes from age-matched wild type (n = 5),* tp53 m/m *(n = 5),* brca2 m/m* (n = 5), and* brca2 m/m;tp53 m/m *(n = 5) male zebrafish (Figure 1(g)). Because testes from* brca2 m/m *and* brca2 m/m;tp53 m/m* zebrafish do not contain spermatozoa, testicular tubules are generally smaller and closer together than in testes from wild type and* tp53 m/m* zebrafish (Figure 1(g)). We therefore compared the numbers of spermatogonia between genotypic groups with complete spermatogenesis (wild type and* tp53 m/m*) and between genotypic groups with arrested spermatogenesis (*brca2 m/m *and* brca2 m/m;tp53 m/m*) (Figure 1(h) and Table S1).

In mammals [36] and zebrafish [33], spermatogonia can be identified as type A or type B based on nuclear morphology, with type A representing a less differentiated population than type B.* tp53 m/m* testes exhibited a significantly increased number of spermatogonia compared to wild type testes that was attributable to expansion of the type A spermatogonial population (p < 0.0001, unpaired t-test; Figure 1(h) and Table S1).* brca2 m/m;tp53 m/m *testes exhibited a significantly increased number of spermatogonia compared to* brca2 m/m* testes. This increase was largely attributable to expansion of the type A spermatogonial population, although the type B spermatogonial populations were also significantly increased (type A, p < 0.0001, unpaired t-test; type B, p = 0.0061, unpaired t-test; Figure 1(h) and Table S1). In* brca2 m/m;tp53 m/m* testes, we observed occasional giant spermatogonia (Figure S2C) and germ cells that were morphologically consistent with perinucleolar oocytes (Figure S2D). We did not observe these cell types in any other genotypic group. The stromal component of the testes (containing various types of somatic cells) appeared similar between zebrafish of different genotypes (Figure 1(g)).

### 3.2. *brca2* Mutation Does Not Alter Ploidy or Cell Cycle Progression in Nonneoplastic Zebrafish Somatic Cells

In preparation for analyzing ploidy in zebrafish cancers, we collected matched nonneoplastic somatic tissue from each cancer-bearing zebrafish. This enabled us to define the diploid population for calculation of tumor ploidy for each cancer specimen and also allowed us to assess the impact of* brca2* mutation on DNA content and cell cycle progression in nonneoplastic somatic cells. We analyzed somatic tissues from 49* brca2 m/m;tp53 m/m* zebrafish and 50* tp53 m/m* zebrafish for DNA content (Table 1 and Figure S2B). 46 tissue samples from* brca2 m/m;tp53 m/m* zebrafish and 49 samples from* tp53 m/m* zebrafish exhibited a single diploid cell cycle. Four nonneoplastic somatic tissues exhibited a small aneuploid peak (ranging from 8.8 to 16.0% of gated cells) in addition to a predominant diploid cell cycle. These tissues were derived from three* brca2 m/m;tp53 m/m* zebrafish and one* tp53 m/m* zebrafish.

**Figure 2:**
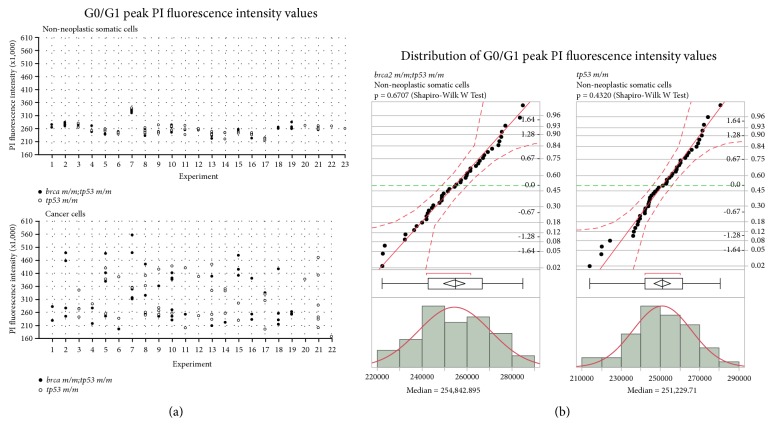
*brca2* mutation does not alter DNA content of nonneoplastic somatic zebrafish tissue. (a) Comparison of G0/G1 peak propidium iodide (PI) fluorescence intensity values for nonneoplastic somatic cells (upper panel) and cancer cells (lower panel) derived from 49* brca2 m/m;tp53 m/m* (black circles) and 50* tp53 m/m* (white circles) cancer-bearing zebrafish. Each circle indicates the G0/G1 peak value for a single sample. Two matched samples (nonneoplastic somatic cells and cancer cells) were analyzed from each individual zebrafish. For every individual zebrafish, the matched nonneoplastic somatic cell sample and cancer cell sample were analyzed in the same experiment. The experiment number indicated on the* x*-axis refers to each independent cell cycle analysis. In experiment 23, the cancer cell sample was excluded (see Materials and Methods), and the G0/G1 peak is only reported for the matched nonneoplastic somatic cell sample. (b) Normal distribution of G0/G1 peak PI fluorescence intensity values for nonneoplastic somatic cells derived from* brca2 m/m;tp53 m/m* and* tp53 m/m* zebrafish. In the normal quantile plot, filled black circles represent individual data points and dashed red lines indicate the Lilliefores confidence bounds. In the outlier box plot, the vertical line represents the median sample value; the diamond contains the mean and upper and lower 95% of the mean; the box ends represent the 25^th^ and 75^th^ quantiles; the whiskers extend to the outermost data points; and the red bracket indicates the shortest half (most dense 50% of observations). In the histogram, vertical bars represent G0/G1 peak intensity values by bin and the overlying red curve fits a smooth curve using nonparametric density estimation.

Comparison of the individual diploid G0/G1 peak propidium iodide (PI) fluorescence intensity values for nonneoplastic somatic cells demonstrated tight clustering of individual values within and between each independent flow cytometry analysis, designated as Experiments 1–23 (Figure 2(a), upper panel). Samples analyzed in one flow cytometry analysis (Experiment 7) showed anomalously high G0/G1 peak PI fluorescence intensity values compared to other samples but were highly similar to one another. Overall, the distributions of G0/G1 peak PI fluorescence intensity values were similar for nonneoplastic somatic cells derived from* brca2 m/m;tp53 m/m* and* tp53 m/m* zebrafish, both within and between each independent flow cytometry analysis. In contrast, the distributions of G0/G1 peak PI fluorescence intensity values for cancer cells derived from* brca2 m/m;tp53 m/m* and* tp53 m/m* zebrafish were highly variable (Figure 2(a), lower panel).

We analyzed the individual G0/G1 peak PI fluorescence intensity values for nonneoplastic somatic cells derived from* brca2 m/m;tp53 m/m* and* tp53 m/m* zebrafish for a normal distribution (Figure 2(b)). For both genotypic groups, the G0/G1 peak PI fluorescence intensity values followed a normal distribution, and the median G0/G1 peak PI fluorescence intensity values were similar (Figure 2(b)).

We assessed cell cycle progression in nonneoplastic somatic tissues based on DNA content distribution by comparing the percent gated cells in G0/G1, S, and G2/M phases (Figure S3). The mean percentages of cells in G0/G1, S, and G2/M phases were similar for* brca2 m/m;tp53 m/m* and* tp53 m/m* zebrafish (Figure S3A).

### 3.3. Tumor Ploidy Is Not Significantly Different in* brca2*-Associated and Non-*brca2*-Associated Zebrafish Cancers

**Figure 3:**
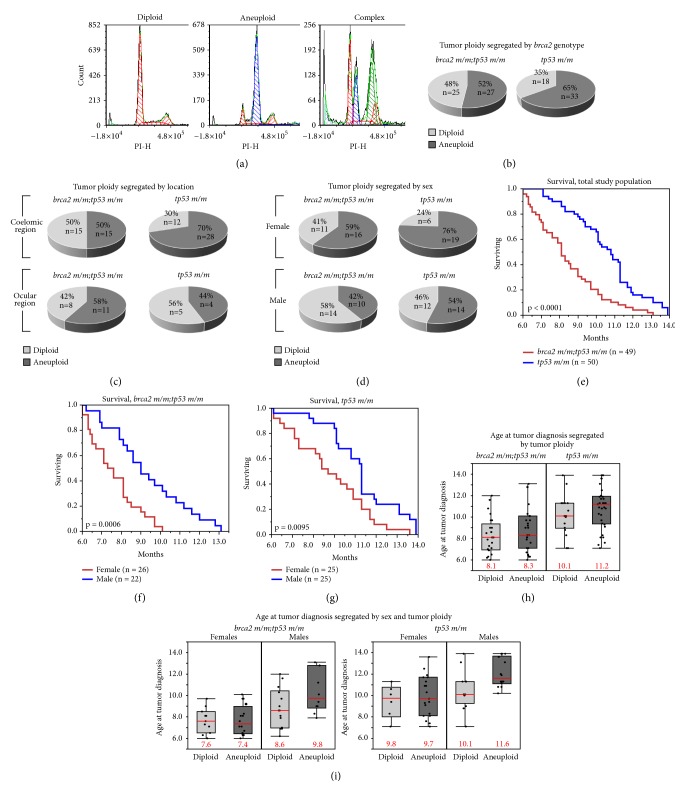
*brca2* mutation status and sex alter ploidy outcomes in zebrafish cancers. (a) Representative propidium iodide (PI) fluorescence histograms from cancers that are diploid or aneuploid or exhibit complex aneuploidy. Software-identified diploid populations are depicted in red; aneuploid populations are depicted in blue and green. ((b)–(d)) Ploidy outcomes segregated by* brca2* genotype alone (b) and in combination with tumor location (c) or sex (d). ((e)–(g)) Kaplan-Meier survival curves for the total study population (e), the* brca2 m/m;tp53 m/m* cohort (f), and the* tp53 m/m* cohort (g). (h) Distribution of ages at tumor diagnosis segregated by* brca2* genotype and tumor ploidy. Median ages at tumor diagnosis are indicated by a red bar and are shown in red text. (i) Distribution of ages at tumor diagnosis segregated by* brca2* genotype, sex, and tumor ploidy. Median ages at tumor diagnosis are indicated by a red bar and shown in red text.

We performed DNA content analysis on cancers derived from 49* brca2 m/m;tp53 m/m* zebrafish and 50* tp53 m/m* zebrafish siblings (Figures 3(a)–3(d) and Table 1). Cancer-bearing zebrafish in these studies were siblings that were distinguished by presence or absence of the* brca2* mutation. Therefore, cancers arising in* brca2 m/m;tp53 m/m* zebrafish are referred to as* brca2*-associated cancers and cancers arising in* tp53 m/m *zebrafish are referred to as non-*brca2*-associated cancers. Cancers were predominantly soft tissue sarcomas that showed variable histologic differentiation toward malignant peripheral nerve sheath tumor (MPNST), as we have previously described in zebrafish of these genotypes [31]. We have previously shown that* brca2* genotype was not correlated to degree of histologic differentiation (poorly differentiated sarcoma versus well-differentiated MPNST) [31]. In comparison, we have previously shown that* brca2 m/m* zebrafish without concurrent* tp53* mutation exhibit a relative increase in the incidence of benign testicular tumors [23, 30].

For each cancer-bearing zebrafish, matched nonneoplastic somatic tissues were simultaneously analyzed as described above. In three* brca2 m/m;tp53 m/m* zebrafish and one* tp53 m/m* zebrafish, there were two anatomically distinct tumors (e.g., both an ocular and a coelomic tumor in one individual) that were collected and analyzed independently. In total, 52 cancers from* brca2 m/m;tp53 m/m* zebrafish and 51 cancers from* tp53 m/m* zebrafish were analyzed. Ploidy was determined by calculating the DNA index based on G0/G1 peak PI fluorescence intensity values (Table S2 and Methods).

Based on DNA index, zebrafish cancers were classified as diploid or aneuploid, and aneuploid tumors were further categorized by type of aneuploidy (Table S2 and Table 2).* brca2* genotype influenced the relative proportions of cancers in diploid and aneuploid categories (Figure 3(b) and Table 2). For* brca2*-associated cancers, approximately equal proportions of cancers were diploid versus aneuploid (48% and 52%, respectively). In contrast, for non-*brca2*-associated cancers the proportion of diploid cancers was almost half the proportion of aneuploid cancers (35% and 65%, respectively). However, the association between* brca2* genotype and ploidy outcome (diploid versus aneuploid) was not statistically significant (p = 0.1877, Chi-square test). For both* brca2*-associated and non-*brca2*-associated cancers, hyperdiploid aneuploidy was the most common nondiploid categorization (Table 2).

Four zebrafish (three* brca2 m/m;tp53 m/m* and one* tp53 m/m* zebrafish) developed two cancers in anatomically distinct locations that were analyzed independently (Figure S4). In three of four individuals, the two cancers did not exhibit the same ploidy (aneuploid versus diploid). In one of four individuals, the two cancers exhibited the same ploidy (both aneuploid).

We assessed cell cycle progression in zebrafish cancers based on DNA content distribution (Figure S3). The mean percentage of cells in G0/G1, S, and G2/M phases were similar for diploid cancers from* brca2 m/m;tp53 m/m* and* tp53 m/m* zebrafish (Figure S3B) and for aneuploid cancers from* brca2 m/m;tp53 m/m* and* tp53 m/m* zebrafish (Figure S3C).

### 3.4. Sex, But Not Tumor Location, Significantly Influences Ploidy Outcome in Zebrafish Cancers

We have previously shown that the coelom and ocular region are the most common sites for cancer development in zebrafish with* brca2* and* tp53* mutations [23, 31], similar to the tp53^M214K^ line [29]. In the current study populations, the majority of analyzed cancer specimens arose in coelomic or ocular locations, with a small number arising in other locations (Table 1). We observed approximately twice as many ocular cancers in* brca2 m/m;tp53 m/m* zebrafish as occurred in* tp53 m/m* zebrafish (Table 1). The association between* brca2* genotype and tumor location (coelom or ocular region) was statistically significant (p = 0.0241, Chi-square test).

To determine if the site of tumor origin influenced ploidy outcome, we assessed the numbers of cancers in each ploidy category arising in coelomic versus ocular locations (Figure 3(c)). Because* brca2* genotype significantly influenced tumor location, ploidy outcomes were assessed only within genotypic groups. For cancers arising in the coelomic region,* brca2*-associated cancers were classified as diploid or aneuploid in equal proportions (n = 15, 50%, for both categories). Non-*brca2*-associated coelomic cancers were predominantly classified as aneuploid (n = 27, 68%). These outcomes were similar to the ploidy outcomes for each genotypic group (Figure 3(b)). For cancers arising in the ocular region, both* brca2*-associated and non-*brca2*-associated cancers were classified as diploid or aneuploid in relatively similar proportions. There was no statistically significant association between location and ploidy outcome (diploid versus aneuploid) for either genotypic group (*brca2 m/m;tp53 m/m*, p = 0.5890;* tp53 m/m*, p = 0. 0.1545; Chi-square test).

To determine the effect of sex on ploidy outcome in zebrafish cancers, we assessed the numbers of cancers in each ploidy category for male and female zebrafish (Figure 3(d) and Table 2). The numbers of males and females were similar in each genotypic group (Table 1) and there was no statistically significant association between* brca2* genotype and sex (p = 0.6920, Chi-square test). Therefore, ploidy outcomes were assessed within genotypic groups and within the entire study population. In both* brca2 m/m;tp53 m/m* and* tp53 m/m* cohorts, the proportions of aneuploid cancers were higher in females than in males of the same genotype (Figure 3(d)). In the* brca2 m/m;tp53 m/m* cohort, 59% of cancers in females were aneuploid (n = 16) versus 42% in males (n = 10). In the* tp53 m/m* cohort, 76% of cancers in females were aneuploid (n = 19) versus 54% in males (n = 14). Despite these differences, there was no statistically significant association between sex and ploidy outcome (diploid versus aneuploid) within genotypic groups (*brca2 m/m;tp53 m/m*, p = 0.2085;* tp53 m/m*, p = 0. 0955; Chi-square test). However, assessment of the entire study population without segregation by* brca2* genotype revealed that the association between sex and ploidy outcome was statistically significant (p = 0.0477), with aneuploid cancers occurring more frequently in female zebrafish (Table 2).

### 3.5. *brca2* Genotype, Sex, Tumor Ploidy, and Survival Outcome Are Interrelated in Cancer-Bearing Zebrafish

We have previously shown that age at tumor diagnosis is statistically significantly lower in* brca2 m/m;tp53 m/m* zebrafish compared to* tp53 m/m* zebrafish [23]. This finding is consistent with survival outcomes in the current study population, which indicated that* brca2* mutation significantly decreases survival time (Figure 3(e), Figures S5A and B, and Table S3). The median age at tumor diagnosis was 8.2 months for* brca2 m/m;tp53 m/m* zebrafish and 10.8 months for* tp53 m/m* zebrafish (Table 1).

Given the difference in ploidy outcomes between male and female zebrafish (Figure 3(d)), we evaluated survival outcomes in male and female cohorts within each genotypic group (Figures 3(f) and 3(g)). Because* brca2* genotype significantly impacted age at tumor diagnosis, survival outcomes were assessed only within genotypic groups. In both* brca2 m/m;tp53 m/m* and* tp53 m/m* zebrafish cohorts, females had a lower median age at tumor diagnosis than males (Table 1), and survival times for females were significantly lower (Figures 3(f) and 3(g), and Table S3).

In human cancers, aneuploidy is often a negative prognostic factor associated with decreased survival time [17–22]. Therefore, we looked for an association between survival time and ploidy outcome for both* brca2 m/m;tp53 m/m* and* tp53 m/m* zebrafish cohorts. In* brca2 m/m;tp53 m/m* zebrafish, the median age at tumor diagnosis was similar for zebrafish with diploid versus aneuploid cancers (Figure 3(h)). In* tp53 m/m* zebrafish, the median age at tumor diagnosis was lower for zebrafish with diploid versus aneuploid cancers (Figure 3(h)). There was no statistically significant difference in survival time for zebrafish with diploid versus aneuploid cancers in either genotypic group (Figures S5C and D and Table S3).

Next, we evaluated for an association between survival time, sex, and ploidy outcome for both* brca2 m/m;tp53 m/m* and* tp53 m/m* zebrafish cohorts. In both* brca2 m/m;tp53 m/m* and* tp53 m/m* zebrafish female cohorts, the median age at tumor diagnosis was similar regardless of ploidy status (Figure 3(i)). In contrast, in both* brca2 m/m;tp53 m/m* and* tp53 m/m* zebrafish male cohorts, the median age at tumor diagnosis was lower for male zebrafish with diploid cancers (Figure 3(i)). We evaluated survival time among individuals of the same sex and genotype that developed diploid versus aneuploid cancers. Within each subgroup of the same genotype and sex, there was no statistically significant difference in survival time based on tumor ploidy (Figures S5E-H and Table S3).

**Figure 4:**
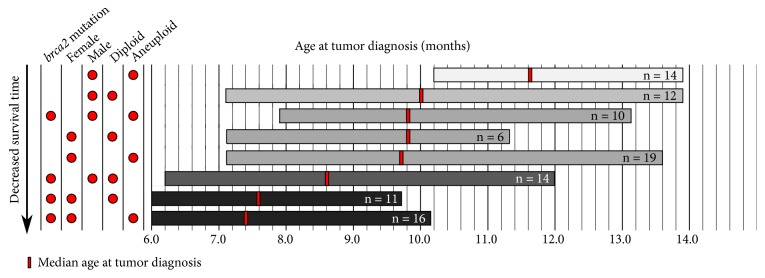
Factors contributing to decreased survival time in cancer-bearing zebrafish. Cohorts are represented by horizontal bars and the numbers of individual zebrafish per cohort are indicated. The horizontal width of each bar shows the range of ages at tumor diagnosis within each cohort, and the vertical red line indicates the median age at tumor diagnosis for each cohort. The combination of filled red circles shown to the left of each horizontal bar indicates the defining characteristics of the cohort with regard to* brca2* mutation status, sex, and tumor ploidy. Cohorts are distributed vertically by decreasing survival time, and color range of horizontal bars from light grey to black reflects longest to shortest survival time, respectively.

We identified two independent variables,* brca2* genotype and sex, that significantly impacted outcomes in this study. Both* brca2* mutation and female sex were associated with significantly decreased survival time, and female sex was associated with a significantly increased proportion of aneuploid cancers. Combined evaluation of* brca2* genotype, sex, and tumor ploidy did not identify a significant interaction among these three variables (Table S4). However, the three groups with the lowest median ages at tumor diagnosis were defined by a combination of (1)* brca2* mutation and female sex (either diploid or aneuploid status) or (2)* brca2* mutation and diploid status (either male or female sex) (Figure 4). These data suggest that zebrafish with these combined variables experience a decrease in survival time.

## 4. Discussion

Genomic instability is a hallmark of cancer cells and a critical contributor to the ongoing genetic evolution that accompanies malignant progression. Chromosomal instability (CIN) is one of the most common forms of genomic instability identified in cancer cells and contributes to the development of both structural aberrations (e.g., rearrangements, amplifications, and deletions) and numerical aberrations (aneuploidy) [37, 38]. Aneuploidy can be an indication of ongoing CIN in cancer cells [39, 40] and is often a negative prognostic indicator in humans with cancer [17–22]. However, this may not be a universal paradigm [16]. Aneuploidy may represent a stable state in cancer cells and does not necessarily indicate ongoing CIN [22, 40–43]. It has also been proposed that aneuploidy may itself induce CIN and thus contribute to a progressively greater level of aneuploidy in cancers [44, 45]. Thus, the development of aneuploidy in cancer cells may be both a cause and consequence of genomic instability in cancer.

The tumor suppressor BRCA2 participates in multiple pathways during both meiosis and mitosis that are critical for maintaining genomic integrity, and loss of BRCA2 function can lead to both structural aberrations and aneuploidy [2]. In this study, we used a* brca2*-mutant/*tp53*-mutant zebrafish line to investigate the impact of* brca2* mutation on cell cycle progression and ploidy outcome in normal tissue (testicular germ cells and somatic cells) and cancers. These heritable* brca2* and* tp53* mutations in zebrafish are similar in location and type to pathologic* BRCA2* and* TP53* mutations in humans [23, 29]. We have previously demonstrated genetic similarities between* BRCA2*-associated human and zebrafish cancers, such as the collaborative effect of* TP53* mutation on carcinogenesis and the loss of heterozygosity in cancer cells [23, 30].

Given* BRCA2*'s role in the resolution of DNA breaks generated during prophase I of meiosis I [3, 4], we first assessed nonneoplastic germ cells from adult zebrafish. As the large size of zebrafish oocytes precludes analysis by flow cytometry, we focused these studies on zebrafish testes. We and others have shown that* Brca2* is expressed in spermatogonia and spermatocytes in vertebrate testes [3, 23, 46] and that testes from* Brca2*-mutant animals exhibit arrested spermatogenesis [3, 4, 23]. In the current study, testes from* brca2 m/m* zebrafish showed an accumulation of cells with 4C DNA content, indicating arrest in meiosis I before completion of the first meiotic cell division. These findings are consistent with previous studies of meiotic progression in* Brca2*-deficient mouse testes [3, 4]. We identified a significantly increased proportion of cells in S-phase from* brca2*-mutant testes. This finding could reflect cell cycle delay; alternatively, it is possible that some cells categorized as S-phase were aneuploid, with DNA content between 2C and 4C. Additional studies will be required to distinguish between these possibilities.

Studies in* Tp53*-deficient mouse models and* Drosophila* have identified a physiologic function for p53 during mitosis and meiosis in gonads [47–50]. In mammalian and zebrafish testes, the mitotic phase of spermatogenesis encompasses development of type A and type B spermatogonia, with type A representing a less differentiated population than type B [33, 36]. These mitotic germ cells undergo both proliferation and differentiation before entering meiosis I as preleptotene spermatocytes [51]. In* Tp53*-deficient mice, type A spermatogonia are significantly increased [47]; spermatogonia are also increased in* tp53*-deficient* Drosophila* [48]. Similarly, we observed that* tp53 m/m* zebrafish testes exhibited significant expansion of the type A spermatogonial population, which corresponded to a significant increase in the proportion of cells with 2C DNA content. Spermatogonial expansion in* Tp53*-deficient mice and Drosophila was attributed to the loss of p53-dependent programmed cell death in mitotic germ cells [47, 48]. p53 was also expressed during meiotic recombination in mouse and* Drosophila* germ cells following the induction of double-strand DNA breaks by the topoisomerase Spo11 [47, 50]. However, loss of p53 does not appear to alter meiotic progression in germ cells in the absence of additional stimuli (e.g., ionizing radiation) [47, 50], although meiotic recombination frequency is reduced [50]. Similarly, our data suggests that loss of p53 does not alter meiotic progression in zebrafish testes: the proportions of cells in 1C, 4C, and S-phase compartments were equivalent in* tp53 m/m* testes compared to wild type testes, and* tp53 m/m* testes were histologically normal.

Strikingly, combined mutations in* brca2* and* tp53* resulted in meiotic arrest and a dramatic accumulation of cells with 2C DNA content, correlating to significantly increased numbers of both type A and type B spermatogonia. These data indicate the significant expansion of mitotic germ cells in* brca2 m/m;tp53 m/m* zebrafish testes. The predominance of type A spermatogonia, which are less differentiated than type B spermatogonia, suggests that spermatogonial differentiation is suppressed in* brca2 m/m;tp53 m/m* zebrafish testes. These outcomes are distinct from the effects of* brca2* or* tp53* mutations alone in zebrafish testes, which caused meiotic arrest or selective type A spermatogonial expansion, respectively. We have not identified a similar effect in published studies of* Brca2*-mutant;*Tp53*-mutant mouse models; a synergistic suppressive effect on germ cell expansion during the initiation of meiosis has been described in testes from juvenile mice with combined mutations in* Brca2* and* Palb2* [4]. However, combined mutations in* tp53* and* rad54* have been shown to alter germ cell numbers in* Drosophila* ovary [50]. Rad54 functions downstream of Brca2 and is essential for homology-directed recombination and DNA repair [52].* Drosophila* ovaries with combined* tp53* and* rad54* mutations showed a variable, frequently increased number of mitotic germ cells (known as nurse cells) [50], which is comparable to the outcome we observed in* brca2 m/m;tp53 m/m* zebrafish testes.

The above-described effects of combined* brca2* and* tp53* mutations on mitotic germ cells (spermatogonia) in zebrafish suggest the interesting possibility that concurrent mutations in* BRCA2* and* TP53* could synergistically promote proliferation and suppress differentiation, which has important implications in the context of cancer initiation. Spermatogenesis is considered to be a classical stem cell-driven process, providing a model for analyzing stem cell physiology and behavior that may be applicable to stem cell populations in other tissues [53, 54]. It is possible that tissue stem and progenitor cells in other sites, which are potential sources for the emergence of cancer stem cells, may be similarly affected by combined* BRCA2* and* TP53* mutations. In support of this concept,* BRCA1* mutation or knockdown has been linked to increased stem/progenitor cell populations and dedifferentiation of stem cells in human breast and mouse mammary tissues [55, 56]. Also, women with* BRCA1*- or* BRCA2*-associated cancer had an increased frequency of breast stem cells in noncancerous breast tissue, which were identified by expression of the stem and progenitor cell marker ALDH [57]. P53 has a known role in the maintenance and regulation of both embryonic and adult stem cells, and wild type p53 suppresses self-renewal and induces differentiation of stem cells after DNA damage (reviewed by Aloni-Grinstein R et al.) [58]. Additionally, proliferation is increased in p53-deficient stem and progenitor cells [59–61]. Together these studies suggest that further investigation of a potentially synergistic role for* BRCA2* and* TP53* mutations in disrupting stem and progenitor cell homeostasis may provide new insight into how mutations in these genes modulate carcinogenesis.

Next, we assessed nonneoplastic somatic tissues and cancers from adult zebrafish. DNA content analysis of nonneoplastic somatic tissues from zebrafish indicated that* brca2* mutation does not alter ploidy in these cells. This outcome is similar to what has been observed in mammalian cells, namely, that normal, nonneoplastic cells generally do not tolerate aneuploidy [37, 43, 44, 62]. We identified a small percentage of aneuploid cells in four somatic tissue samples but cannot rule out the possibility that these tissues contained early-stage cancers not detectable by stereomicroscopic examination. In zebrafish cancers, we observed that diploidy was more common in* brca2*-associated cancers than non-*brca2*-associated cancers, although this difference was not statistically significant. In comparison, diploid and aneuploid cancers reportedly occur in roughly similar proportions in human* BRCA2*-associated and non-*BRCA2*-associated cancers [14–16]. Contrastingly, aneuploidy and polyploidy were increased in* Brca2-*inactivated tumor cell lines derived from a mouse model of pancreatic ductular adenocarcinoma [63]. Overall, our data from* brca2*-associated zebrafish cancers parallels previous reports that* BRCA2* mutation does not significantly increase the rate of aneuploidy in human cancers.

We additionally considered sex as a variable that might influence tumor ploidy in zebrafish. Aneuploidy was significantly more common in female zebrafish in the full study population, although significance was not maintained when study cohorts were segregated by* brca2* genotype. To our knowledge, the impact of sex on tumor ploidy has not been previously reported in zebrafish cancer models. In humans, gender is not linked to the development of global numerical aberrations in cancers, although numerical aberrations specifically affecting the sex chromosomes (gonosomes) occur more frequently in cancers from males [64]. However, gender-specific structural aberrations affecting both gonosomes and autosomes are reported in some cancer types that may be biologically and prognostically significant [64–67]. The factors that drive accumulation of these gender-associated genomic changes are not yet defined. Our zebrafish model will be an informative tool for investigating how sex impacts the accumulation of genetic and genomic changes during carcinogenesis.

Finally, we evaluated survival outcome in cancer-bearing zebrafish in the context of the three major variables analyzed in this study (*brca2* genotype, sex, and tumor ploidy). We have previously reported that* brca2*-mutant zebrafish develop tumors at a significantly younger age than non-*brca2*-mutant zebrafish [23], which was also observed in this study. We additionally identified a significant impact of sex on survival outcome: females in both genotypic groups developed tumors at a statistically significant younger age than males. We are unaware of any previous report of a zebrafish cancer model that experiences a significant disparity in survival outcome based on sex. In humans, survival outcomes in females are generally better than in males [68]. However, there are some cancer types for which survival outcomes are reversed; i.e. survival outcomes in women are worse than in men [69, 70]; such differences have also been linked to response to targeted therapies [71]. Gender-associated differences in survival outcome have yet to be explained in humans, although multiple possible contributors, including hormonal signaling, environmental exposures, DNA repair defects, and other factors, have been postulated [65, 70]. The potential contributions of such factors to carcinogenesis in our zebrafish model are not yet known.

Ploidy is an independent prognostic factor for survival across a variety of human cancer types, with aneuploidy associated with worse prognosis [17–22]. Within both genotypic groups of cancer-bearing zebrafish in this study, we found that median survival times in females with diploid versus aneuploid cancers were similar, while median survival times for males with diploid cancers were lower than for males with aneuploid cancers. This finding is surprising, given that aneuploidy is generally linked to worse prognosis in human cancer patients. However, diploidy has been correlated to worse prognosis in* BRCA2*-associated human breast cancers [16]. In our study, ploidy did not emerge as a variable that significantly contributed to survival outcome in cancer-bearing zebrafish. Since we identified both* brca2* genotype and sex as variables that significantly influenced survival outcome, we could not assess the impact of ploidy on survival outcome independently from these variables. As a result, we cannot rule out the possibility that ploidy would have been found to significantly affect survival outcome in a larger study population. In comparison, the aforementioned study of ploidy status and survival outcome in human breast cancer patients with and without* BRCA2* mutation presented data from almost 3,000 patients that was acquired over a 50-year period [16].

We observed the lowest median survival times in zebrafish with (1)* brca2* mutation and female sex and (2)* brca2* mutation and diploid cancer (Figure 4). Although diploidy is typically linked to relative genomic stability, diploid cancers may actually be “pseudodiploid,” exhibiting complex genomic alterations that do not impact total chromosomal content. This condition has been described in diploid* BRCA2*-associated human cancers [15] and was proposed as a contributor to poor prognosis in patients with diploid* BRCA2*-associated cancers [16]. Similarly, near-diploid colorectal cancers have been shown to possess extensive genomic changes that may be essential in carcinogenesis [72]. On the other hand, not all aneuploid cancers experience ongoing CIN but rather exhibit relative genomic stability [22, 40–43]. It is therefore possible that, in our model system, diploid or aneuploid categorizations do not reflect the level of genomic stability in these zebrafish cancers. Further studies are underway to investigate more deeply the genetic and genomic alterations that characterize cancers in our zebrafish model and determine the impact of* brca2* mutation and sex on these alterations.

## 5. Conclusions

Our findings confirm that the individual effects of* brca2* and* tp53* mutations on testicular germ cell development are conserved in zebrafish and reveal that combined* brca2* and* tp53* mutations collaborate to promote accumulation of spermatogonia while suppressing spermatogonial differentiation. Our findings additionally identify both* brca2* genotype and sex as independent variables that significantly affect survival outcome in cancer-bearing zebrafish. While ploidy outcome in zebrafish cancers did not significantly affect survival outcome, ploidy was significantly influenced by sex. Finally, we determined that diploidy is not linked to better survival outcome in cancer-bearing zebrafish: the worst survival outcomes were observed with (1)* brca2* mutation and female sex and (2)* brca2* mutation and diploid cancer. These studies provide new insight into the impact of combined* BRCA2* and* TP53* mutations on germ cell development and identify key influences of* BRCA2* mutation, sex, and ploidy on survival outcome in vertebrate cancer.

## Figures and Tables

**Table 1:** Characteristics of the study population used for analysis of somatic and cancer cells.

	*brca2 m/m;tp53 m/m*	*tp53 m;m*
*Total zebrafish*	49	50

Males	22 (45%)	25 (50%)

Females	26 (53%)	25 (50%)

Sex not determined	1 (2%)	0 (0%)

*Total tumors* ^a^	52	51

*Age at tumor diagnosis (mo)*		

Median age (total population)	8.2	10.8

Range (total population)	6.0 – 13.1	7.1 – 13.9

Median age (males)	9.0	11.3

Range (males)	6.2 – 13.1	7.1 – 13.9

Median age (females)	7.4	9.7

Range (females)	6.0 – 10.1	7.1 – 13.6

*Tumor location*		

Coelom	30 (58%)	40 (78%)

Ocular region	19 (37%)	9 (18%)

Other	3 (6%)	2 (4%)

^a^ Two anatomically distinct tumors were independently analyzed for three *brca2 m/m;tp53 m/m *zebrafish and one* tp53 m/m *zebrafish.

**Table 2:** Impact of *brca2* genotype and sex^a^ on the relative proportions of zebrafish cancers in diploid and aneuploid categories.

Ploidy category	*brca2 m/m;tp53 m/m*	*tp53 m;m*
Diploid	25 (48%)	18 (35%)

Aneuploid	27 (52%)	33 (65%)

* Hypodiploid aneuploid*	*4*	*3*

* Hyperdiploid aneuploid*	*19*	*23*

* Tetraploid aneuploid*	*1*	*0*

* Complex*	*3*	*7*

Ploidy category^b^	Females	Males

Diploid	17 (33%)	26 (52%)

Aneuploid	35 (67%)	24 (48%)

* Hypodiploid aneuploid*	*4*	*3*

* Hyperdiploid aneuploid*	*25*	*16*

* Tetraploid aneuploid*	*1*	*0*

* Complex*	*5*	*5*

^a^The sex for one zebrafish was not determined and is not included in the comparison of ploidy in females versus males.

^b^Categorization of ploidy outcomes by sex includes all cancer-bearing zebrafish without segregation by *brca2* genotype. Ploidy outcomes segregated by both sex and genotype are presented in Figure 3(d).

## Data Availability

All data generated or analyzed during this study are included in this published article (and its Supplementary Information files). The zebrafish lines described in this work (wild type (AB),* brca2*^*hg5*^ mutant, and* tp53*^*zdf1*^ mutant) are maintained at the Shive laboratory and may be accessed by contacting the corresponding author.

## Supplementary Materials

Supplementary Materials The supplementary materials include Supplementary Figure Legends, Supplementary Figures  S1–S5, and Supplementary Tables  S1–S4. Presented in the Supplementary Figures are the following data: Figure  S1, gating strategies for analysis of flow cytometry data from testes, nonneoplastic somatic cells, and cancer cells; Figure  S2, representative histologic images of testes from* brca2 m/m;tp53 m/m* and* tp53 m/m* zebrafish; Figure  S3, distributions of cells (mean percent of gated cells) according to DNA content in nonneoplastic somatic tissues and cancers; Figure  S4, example of disparate ploidy outcomes from two anatomically distinct cancers derived from the same zebrafish; and, Figure  S5, analysis of survival outcome with respect to various population characteristics (*brca2* mutation status, sex, and tumor ploidy). Presented in the Supplementary Tables are the following data: Table  S1, comparison of DNA content and spermatogonia counts in testes from wild type,* tp53 m/m*;* brca2 m/m*, and* brca2* m/m;tp53 m/m zebrafish; Table  S2, definition of DNA ploidy categories determined by the calculated DNA index; Table  S3, summary of statistical analyses of survival outcomes in cancer-bearing zebrafish; and, Table  S4, summary of statistical analyses assessing the impact of multiple variables on survival outcomes in cancer-bearing zebrafish.
