# Supplementary material for: Genotypic and Phenotypic Variables Affect Meiotic Cell Cycle Progression, Tumor Ploidy, and Cancer-Associated Mortality in a brca2-Mutant Zebrafish Model
Source: J Oncol. 2019 Feb 26;2019:9218251. doi: 10.1155/2019/9218251 (PMC6413366; doi:10.1155/2019/9218251)
Supplement: Supplementary Materials — The supplementary materials include Supplementary Figure Legends, Supplementary Figures S1–S5, and Supplementary Tables S1–S4. Presented in the Supplementary Figures are the following data: Figure S1, gating strategies for analysis of flow cytometry data from testes, nonneoplastic somatic cells, and cancer cells; Figure S2, representative histologic images of testes from brca2 m/m;tp53 m/m and tp53 m/m zebrafish; Figure S3, distributions of cells (mean percent of gated cells) according to DNA content in nonneoplastic somatic tissues and cancers; Figure S4, example of disparate ploidy outcomes from two anatomically distinct cancers derived from the same zebrafish; and, Figure S5, analysis of survival outcome with respect to various population characteristics (brca2 mutation status, sex, and tumor ploidy). Presented in the Supplementary Tables are the following data: Table S1, comparison of DNA content and spermatogonia counts in testes from wild type, tp53 m/m; brca2 m/m, and brca2 m/m;tp53 m/m zebrafish; Table S2, definition of DNA ploidy categories determined by the calculated DNA index; Table S3, summary of statistical analyses of survival outcomes in cancer-bearing zebrafish; and, Table S4, summary of statistical analyses assessing the impact of multiple variables on survival outcomes in cancer-bearing zebrafish. [file 9218251.f1.pdf]

Figure S1

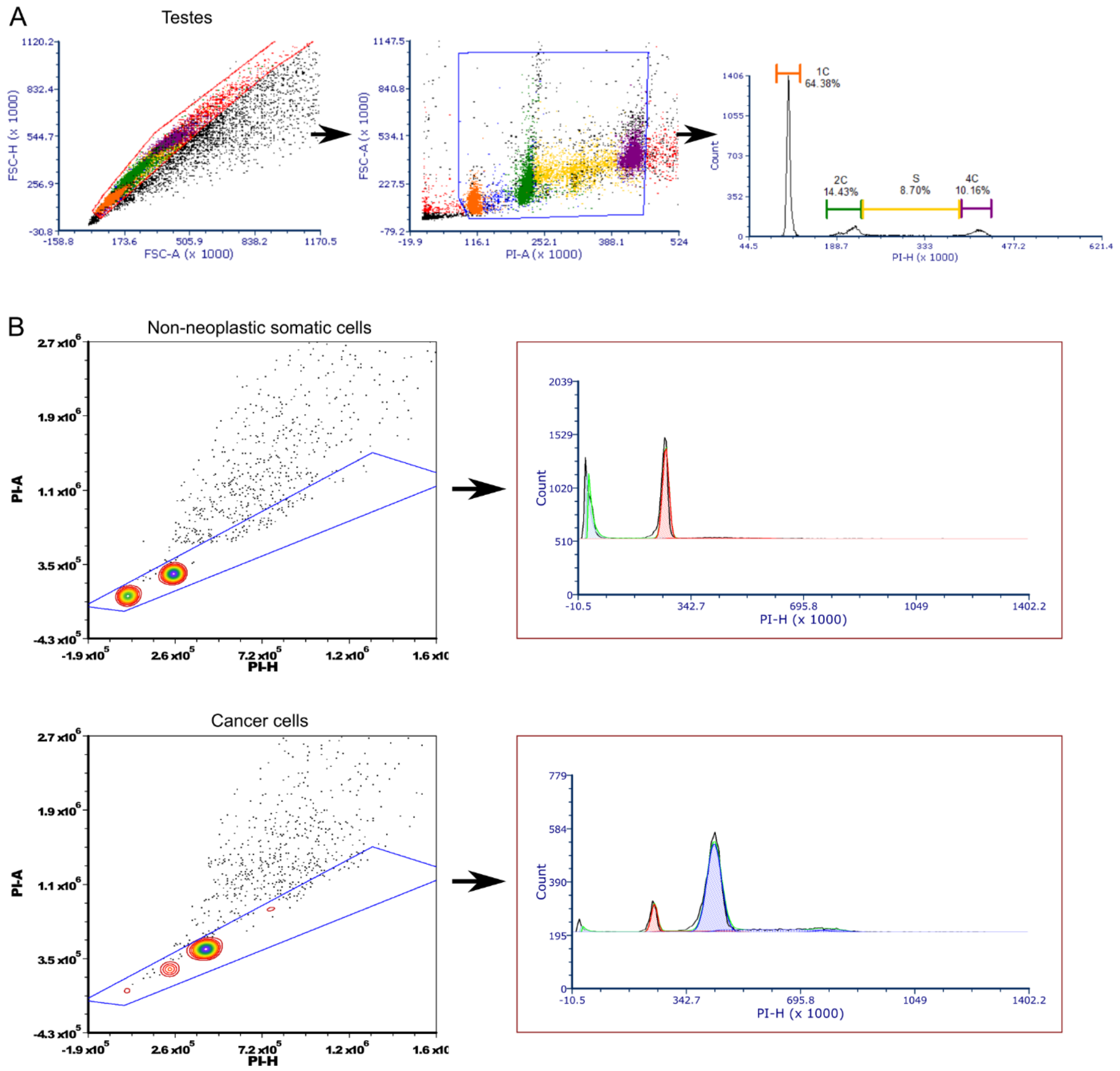

Figure S1. Gating strategies for analysis of flow cytometry data for testes (A) and non-neoplastic somatic and cancer cells (B). A, Gating for doublet discrimination (red gate) and definition of the cell population used to generate the DNA histogram. The 1C, 2C, S, and 4C populations are defined by colored marker gates, and accurate identification of each population can be confirmed by color in the corresponding dot plots. B, Gating for doublet discrimination (blue gate). In the DNA histogram, the green peak represents debris; the red peak represents the software-identified diploid population; and the blue peak represents the software-identified aneuploid population.

Figure S2

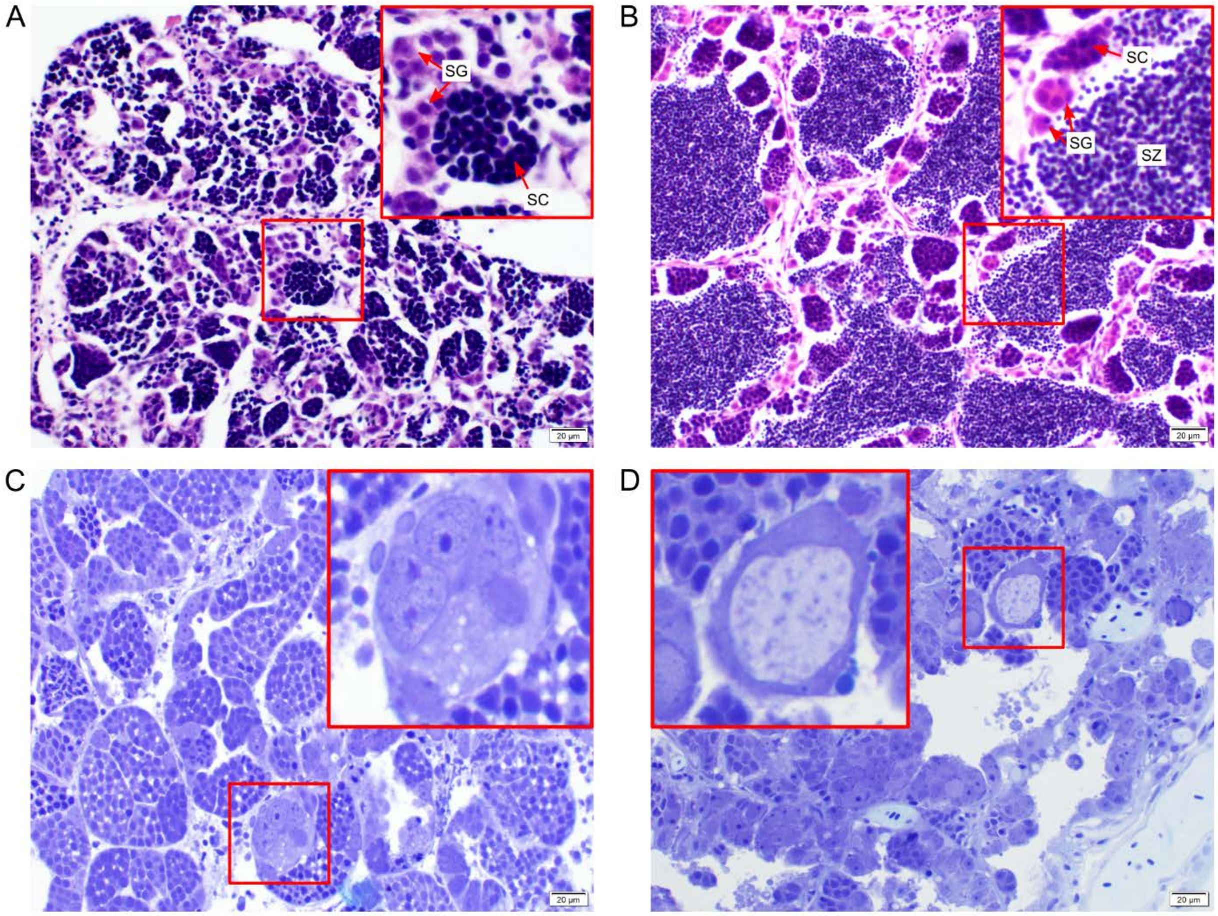

Figure S2. Gonads from *brca2 m/m;tp53 m/m* zebrafish are histologically abnormal. A, Representative histologic image of testes from a *brca2 m/m;tp53 m/m* zebrafish demonstrating arrested spermatogenesis, hematoxylin and eosin (H &E) stain. B, Representative histologic image of testes from a *tp53 m/m* zebrafish demonstrating complete spermatogenesis, H & E stain. SG, spermatogonia; SC, spermatocytes; SZ, spermatozoa. C, Giant spermatogonia in testes from a *brca2 m/m;tp53 m/m* zebrafish, Toluidine blue stain. D, Perinucleolar oocyte in testes from a *brca2 m/m;tp53 m/m* zebrafish, Toluidine blue stain.

Figure S3

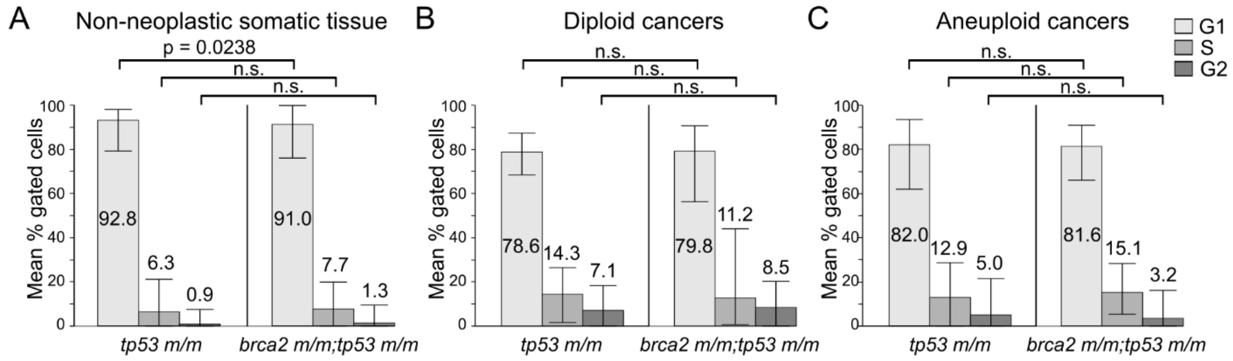

Figure S3. *brca2* mutation status does not alter distributions of cells according to DNA content in non-neoplastic somatic tissue or cancers. Mean percent of gated cells in each DNA content category for non-neoplastic somatic tissue (A), diploid cancers (B), and aneuploid cancers (C), segregated by *brca2* genotype. The mean percent value for each DNA content category is shown. Error bars represent the range of the data.

Figure S4

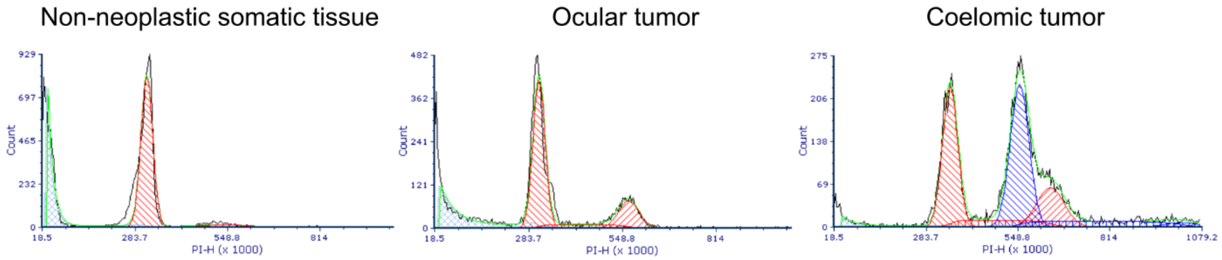

Figure S4. Disparate ploidy outcomes from two anatomically distinct cancers derived from the same zebrafish.

Figure S5

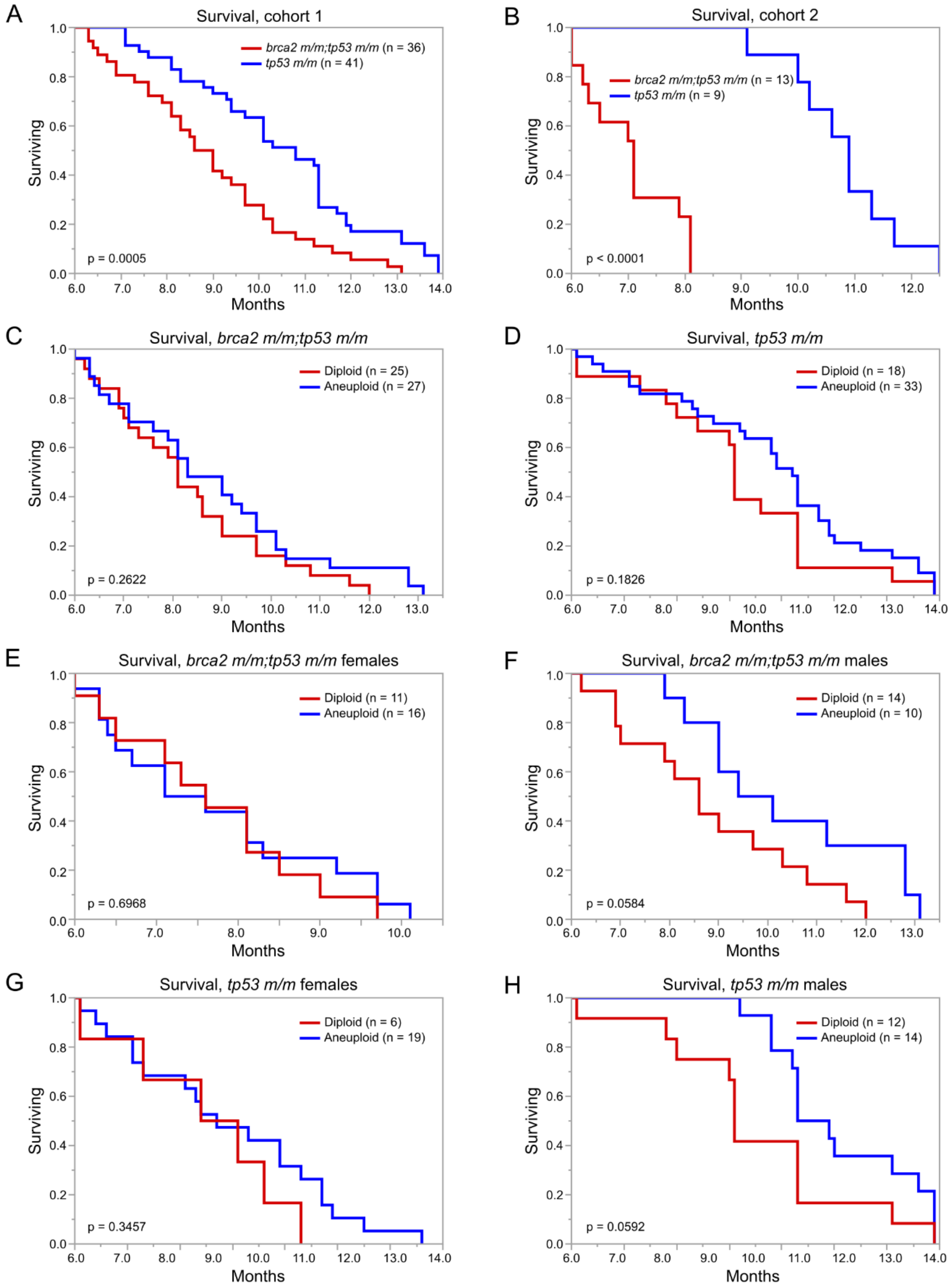

Figure S5. *brca2* mutation status and sex, but not ploidy outcome, significantly impact the age at tumor diagnosis in zebrafish. A and B, Kaplan-Meier survival curves for cohort 1 (A) and

cohort 2 (B) (see Methods). C and D, Kaplan-Meier survival curves for *brca2 m/m;tp53 m/m* (C) and *tp53 m/m* (D) cohorts segregated by ploidy outcome. E and F, Kaplan-Meier survival curves for *brca2 m/m;tp53 m/*m females (E) and males (F) segregated by ploidy outcome. G and H, Kaplan-Meier survival curves for *tp53 m/*m females (G) and males (H) segregated by ploidy outcome.

Table S1. Comparison of DNA content and spermatogonia counts in zebrafish testes.

**Testes with complete spermatogenesis**					
	**Wild type testes**		***tp53 m/m* testes**		
**DNA content**	**Mean (%)**	**Range (%)**	**Mean (%)**	**Range (%)**	**p-value**
1C	68.6	62.9 – 77.4	63.4	48.5 – 74.9	0.2527
2C	13.5	10.1 – 15.2	17.2	14.2 – 22.3	0.0405
S	7.6	5.9 – 9.1	7.1	5.2 – 9.4	0.5592
4C	8.2	4.8 – 11.7	8.7	4.0 – 12.5	0.593
**Spermatogonia count**	**Mean**	**Range**	**Mean**	**Range**	**p-value**
Type A	23	10 – 30	47	20 – 67	< 0.0001
Type B	18	3 – 35	20	12 – 30	0.5749
Total	45	13 – 51	67	36 – 87	< 0.0001

**Testes with incomplete spermatogenesis**					
	***brca2 m/m* testes**		***brca2 m/m;tp53 m/m* testes**		
**DNA content**	**Mean (%)**	**Range (%)**	**Mean (%)**	**Range (%)**	**p-value**
2C	27.1	19.0 – 45.8	58.4	45.6 – 71.7	0.0021
S	20.3	18.0 – 24.4	9.9	6.0 – 14.0	0.0019
4C	44.7	29.5 – 57.1	19.4	11.2 – 26.1	0.0002
**Spermatogonia count**	**Mean**	**Range**	**Mean**	**Range**	**p-value**
Type A	36	14 – 53	124	87 – 215	< 0.0001
Type B	37	15 – 72	54	32 – 79	0.0061
Total	73	29 – 108	178	127 – 277	< 0.0001

Table S2. DNA ploidy definitions calculated from the DNA index.[a]

**DNA index[b]**	**Ploidy category**
< 0.85	Hypodiploid
1.0 ± 0.15	Diploid
> 1.15 and < 1.90	Hyperdiploid aneuploid
> 1.90 and < 2.10	Tetraploid aneuploid
> 2.10	Hypertetraploid aneuploid

[a] Modified from Ross JS et al., Am J Clin Pathol 2003; 120(Suppl1):S72-S84.

[b] DNA index equals the ratio of the tumor G1 peak fluorescence intensity value to a standard DNA fluorescence intensity value. See Materials and Methods for additional details.

Table S3. Summary of statistical analyses of survival outcomes in cancer-bearing zebrafish. Survival outcomes were assessed by the log-rank test and the Cox's Proportional Hazard Model (CPHM).

	**Log-Rank Test**	**CPHM**	**Hazard Ratio**
**Genotype** (*brca2 m/m;tp53 m/m* versus *tp53 m/m*)	< 0.0001	< 0.0001	2.68
**Sex** (female versus male)			
brca2 m/m;tp53 m/m	0.0006	0.0010	2.94
tp53 m/m	0.0095	0.0158	2.06
**Ploidy** (diploid versus aneuploid)			
brca2 m/m;tp53 m/m	0.2622	0.2810	1.36
tp53 m/m	0.1826	0.2329	1.43
**Ploidy in female zebrafish** (diploid versus aneuploid)			
brca2 m/m;tp53 m/m	0.6968	0.7197	1.16
tp53 m/m	0.3457	0.3756	1.57
**Ploidy in male zebrafish** (diploid versus aneuploid)			
brca2 m/m;tp53 m/m	0.0584	0.0620	2.33
tp53 m/m	0.0592	0.1138	1.89

Table S4. Summary of statistical analyses assessing the impact of multiple variables on survival outcomes in cancer-bearing zebrafish. The impact of combined variables on survival outcome was assessed by the Cox's Proportional Hazard Model.

**Total cohort**	**p-value**
Genotype*Ploidy	0.8896
Genotype*Sex	0.2806
Ploidy*Sex	0.3140
Genotype*Ploidy*Sex	0.6259
***brca2 m/m;tp53 m/m* cohort**	**p-value**
Ploidy*Sex	0.2333
***tp53 m/m* cohort**	**p-value**
Ploidy*Sex	0.7482
